# Is clindamycin effective in preventing infectious complications after oral surgery? Systematic review and meta-analysis of randomized controlled trials

**DOI:** 10.1007/s00784-022-04411-2

**Published:** 2022-03-02

**Authors:** Iciar Arteagoitia, Fabio Rodríguez Sánchez, Amaia Figueras, Nagore Arroyo-Lamas

**Affiliations:** 1 Stomatology Department, University of the Basque Country UPV/EHU, 48940 Leioa, Bizkaia Spain; 2 BioCruces Health Research Institute Cruces Plaza, 48903 Barakaldo, Bizkaia Spain; 3 Department of Preventive Medicine and Public Health, University of the Basque Country, UPV/EHU, 48940 Leioa, Bizkaia Spain; 4 Department of Oral Health Sciences, Section Periodontology, Catholic University of Leuven & University Hospitals Leuven, Leuven, Belgium; 5 University of the Basque Country UPV/EHU, Bizkaia 48940 Leioa, Spain; 6 Medicine and Surgery Program, PhD School, University of the Basque Country UPV/EHU, 48940 Leioa, Bizkaia Spain

**Keywords:** Clindamycin, Infection, Oral surgery, Third molar surgery, Antibiotic prophylaxis, Systematic review

## Abstract

**Objective:**

To determine the effect of clindamycin in the prevention of infection after oral surgery.

**Material and Methods:**

This systematic review and meta-analysis followed the PRISMA statement, the PICO-framework and included only randomized controlled clinical trials. In all studies clindamycin was administered to prevent infections in patients who underwent oral surgery. Two independent researchers conducted the search, data extraction and risk of bias assessment. Included studies were classified by the type of oral surgery. Besides, data of patients, procedures and outcome variables were collected. Risk ratios (RR) and 95% confidence intervals (CI) were calculated by using Mantel–Haenszel model and the number needed to treat (NNT). Finally, any potential sources of heterogeneity were estimated.

**Results:**

Seven trials of 540 articles met the inclusion criteria and were included in the qualitative synthesis. Four articles assessing the effect of oral clindamycin in third molar surgery were quantitatively analyzed. The overall RR was 0.66 (95% CI = 0.38–1.16), being non-statistically significant (*p* = 0.15). There was no heterogeneity between the studies I^2^ = 0, p = 0.44. The NNT was 29 (95% CI = 12- -57).

**Conclusions:**

The effectiveness of clindamycin could not be evaluated except in third molar extraction. Oral clindamycin is ineffective in preventing infection in third molar surgery.

**Clinical Relevance:**

There is a lack of high-quality evidence supporting the prescription of clindamycin to prevent infections after oral surgery, despite being frequently prescribed as an alternative for penicillin-allergic patients. Oral clindamycin has not been shown to be effective after third molar extractions.

## Introduction

Despite the recognized economic and public health implications of the indiscriminate use of antibiotics, professionals prescribe very frequently preventive antibiotics in common oral surgeries such as third molar extractions and oral implant placements in healthy patients [1, 2].

Besides, several surveys conducted in different countries have shown that many professionals continue to prescribe preventive antibiotics after different oral surgeries, in order to prevent infectious complication [3–5].

Numerous clinical trials have been carried out to assess the effectiveness of different antibiotic treatments to prevent infection after dental extractions, as we can appreciate in the Cochrane review update of 2021 [6]. Indeed, there is no consensus on the use of antibiotics for preventing surgical infection associated with oral implant placement in healthy patients [7–11].

Penicillin and other antibiotics from its group are the most frequently prescribed in dentistry. Nevertheless, some important questions are brought up relating to patients allergic to them. Clindamycin is widely used in oral surgeres as an alternative preventive treatment in patients allergic to amoxicillin [12–14]. In fact, previous studies reported a remarkable effectiveness of clindamycin in reducing the incidence of infectious and inflammatory complications after third molar surgery such as dry socket [15]. However, recent evidence suggests a lack of benefits [14].

Indeed, despite being commonly used as an alternative in penicillin-allergic patients, the effect of clindamycin on oral surgery has not been yet exactly determined in the current literature [16]. For these reasons, it was considered necessary to perform a systematic review and, if it was possible, to conduct a meta-analysis on this topic.

The aim of this study was to assess the effect of clindamycin (with any kind of route of administration, regimen or dosage) to prevent infectious complications in patients who underwent any type of oral surgery.

## Material and methods

This study was reported in accordance with the Preferred Reporting Items for Systematic Reviews and Meta-Analyses (PRISMA). Prior to conduct the review, its methods were established. The study protocol has been registered, and approved in PROSPERO with the registration number CRD4202122624. It can be accessed on the following link.

https://www.crd.york.ac.uk/prospero/display_record.php?RecordID=226241

The null hypothesis (H0) was tested with a significance level of *p* = 0.05, since the preventive use of clindamycin is not effective in reducing infection in oral surgery.

### Eligibility criteria

Only randomized clinical trials (RCT) controlled with placebo or without any treatment were included, regardless of whether they were double-blinded or not. At least patients from one of the groups must have received preventive clindamycin (with any kind of route of administration, regimen or dosage) to prevent infectious complications after any type of oral surgery procedure. The articles were classified according to the type of oral surgery, in which the effectiveness of clindamycin was tested.

All studies that did not meet the inclusion criteria were excluded, particularly noteworthy are those trials in which the control group received an antibiotic treatment.

### Information sources

The following electronic databases were used for conducting the search: Pubmed/Medline, Cochrane Central Register of Controlled Trials (CENTRAL), Web of Science, Embase Ovid and Scopus. Manual search was also carried out. All databases were searched up to January 2021.

### Search

The search strategy was based on the PICO-framework. Population (P): Patients were assessed for inclusion in the analysis regardless of their age, gender, previous pathologies or habits, such as smoking. All studies evaluating any type of oral surgical procedure were included. Intervention (I): Antibiotic prophylaxis with clindamycin administered orally, intravenously or topically and prescribed before, during and/or after oral surgery. Comparison (C): Placebo or no treatment gave peri-operatively. Outcome (O): The outcome variables included all signs of postoperative infection (pain, fever, swelling, trismus, and wound or surgical site infection), dry socket, other related complications and adverse events. Two independent researchers performed the study selection until January 2021.

The electronic search in the PubMed/Medline database was carried out by using MeSH thesaurus and search algorithms connected with Boolean operators as key words for titles and abstracts. This is one of the different search strategies used: ("clindamycin"[MeSH Terms] OR "clindamycin"[All Fields] OR "clindamycine"[All Fields]) AND ("surgery, oral"[MeSH Terms] OR ("surgery"[All Fields] AND "oral"[All Fields]) OR "oral surgery"[All Fields] OR ("oral"[All Fields] AND "surgery"[All Fields]) OR "oral surgery"[All Fields] OR "oral surgical procedures"[MeSH Terms] OR ("oral"[All Fields] AND "surgical"[All Fields] AND "procedures"[All Fields]) OR "oral surgical procedures"[All Fields] OR ("oral"[All Fields] AND "surgery"[All Fields]).

No restrictions were used on the language or date of publication. The filters activated were: humans and clinical trials.

### Study selection

**Fig. 1:**
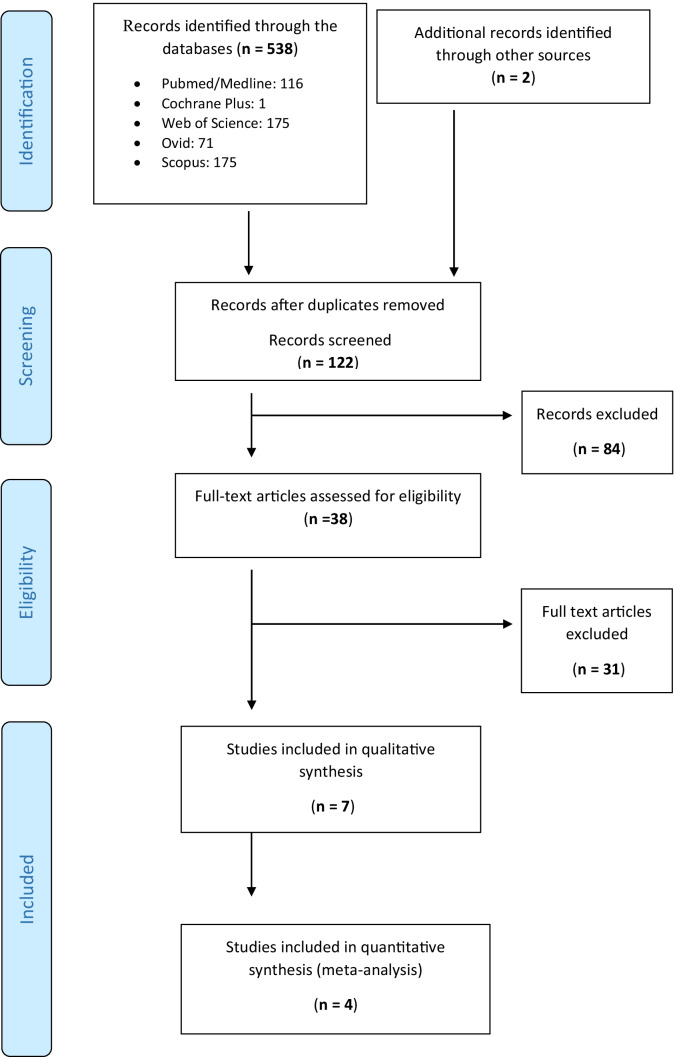
PRISMA flow diagram

The search strategy produced the results shown in Fig. 1. The databases not listed in this figure did not yield any relevant publications. Two independent researchers performed the selection of studies (IA and AF), a third researcher was requested in case of conflict (FR). The included and excluded articles with the reasons for exclusion were recorded in Table 1.

**Table 1:** Full-text articles classified according to the surgical procedure in which clindamycin was tested, specifying those included, excluded and the reason for exclusion

Surgical procedures	Authors/ Year	Inclusion /exclusion
Mandibular fractures	Miles BA, Potter JK, Ellis E 2006 [17]	Excluded: no control group with placebo or without treatment
Bone grafts along with implant placement	Lindeboom JA, 2005 [18]	Excluded: no control group with placebo or without treatment
	Lindeboom JA, 2006 [19]	Excluded: no control group with placebo or without treatment
	Klinge A, Khalil D, Klinge B et all 2020 [20]	Excluded: It is a review
Orthognathic surgery	Lindeboom JA, Baas EM, Kroon FH 2003 [21]	Excluded: no control group with placebo or without treatment
	Baqain ZH, Hyde N, Patrikidou A, Harris M.2004 [22]	Excluded: no control group with placebo or without treatment
	Davis CM, Gregoire CE, Davis I, Steeves TW.2017 [23]	Excluded: no control group with placebo or without treatment
Oncologic surgery	Righi M, Manfredi R, Farneti G, et all 1995 [24]	Excluded: no control group with placebo or without treatment
Head and neck surgery	Mann W, Maurer J, Wolfensberger M, et all 1990 [25]	Excluded: no control group with placebo or without treatment
	Clayman GL, Raad II, Hankins PD et all 1993 [26]	Excluded: no control group with placebo or without treatment
Endodontic procedure	Raslan N, Mansour O, Assfoura L. 2017 [27]	Excluded: no control group with placebo or without treatment
Endodontic surgery	Lindeboom JA, Frenken JW, Valkenburg et all 2005 [28]	Included
Dental extraction	Laird WR 1972 [29]	Excluded: no control group with placebo or without treatment
	Bystedt 1980 [30]	Excluded: did not report data in a form suitable for inclusion
	Kupfer 1995 [15]	Excluded: it is not a RCT
	Poeschl 2003 [31]	Included
	&Foy SP, Shugars DA, Phillips C, et all 2004 [32]	Excluded: did not report data in a form suitable for inclusion
	^a^Halpern LR,0.2007 [12]	Included
	Kaczmarzyk 2007 [33]	Included
	Adde, 2012 [34]	Included
	*Hamiti-Krasniqi 2014 [35]	Included
	Xue 2014 [36]	Excluded: patients included in another study. It was not possible to contact the authors to confirm this
	Xue 2015 [13]	Excluded: did not report data in a form suitable for inclusion
	Kaposvári 2017 [37]	Included

*TOPICAL CLINDAMYCIN; ^a^INTRAVENOUS CLINDAMYCIN

### Data collection process

A data collection protocol was designed, in which each selected study was independently reviewed by two investigators (IA and NAL), and differences were resolved by consulting a third analyst (FR). When there was no explicit data in the main text, calculations were performed using the results in tables or figures, when it was possible. In case of lack or doubt about data of interest in the article, the authors were contacted.

### Data items

Table 2 included all data recorded in each study. Studies were classified according to the type of oral surgery performed. Apart from that, when more than one antibiotic was tested in the same study, only the information regarding the patients who were treated with clindamycin and those who belonged to the control groups was collected.

**Table 2:** Characteristics of the studies included in the review

AUTHOR YEAR COUNTRY Funding	STUDY DESIGN INCLUSIONCRITERIA PATIENTS		ANTIBIOTIC/ PLACEBO		POST-OPERATIVE TREATMENT ANALGESIA		OUTCOME FOLLOW UP PERIOD		RESULTS		LOOSE TO FOLLOW UP SIDE EFFECTS	
ORAL CLINDAMYCIN IN THIRD MOLAR EXODONTIA												
Poeschl [31] 2004 Austria Funding source: unspecified	RCT Surgical extraction of impacted lower third molars Mean age 20.7 years (the age range was between 14 and 61 years)		Experimental group: 300 mg of clindamycin (Dalacin) orally 3 times a day, during 5 days post operation N = 180 molars Control group: nothing. N = 172 molars		Mouth rinse with 0.2% chlorhexidine 1 min before surgery Analgesic after surgery if necessary 500 mg of mefenanimo acid. Every 6 h		Dry socket: lack of clot, exposed bone, necrotic smelly remains in the cavity, extremely painful socket walls Infection of the suture area: local inflammation, hyperemia, purulent exudate and pain in the area of ​​the suture) Pain assessment: VAS scale Differences in mouth opening Follow-up period: day 2, 10 and 4 weeks		Experimental group: local infection symptoms Dry socket 15/180 Control group: local infection symptoms Dry socket 17/172 Not included in the study amoxicillin /clavulanis group		2 patients did not return after surgery 4 patients did not remove the suture on day 10 7 patients did not return for the last appointment at 4 weeks after surgery Side effects: headache, weakness, nausea, tremor, diarrhea, constipation, insomnia, and fever. Experimental group 22 and control group 24	
Kaczmarzyk [33] 2007 Poland Funding source: unspecified	RCT Healthy volunteers, surgical extraction of a retained lower third molar, requiring bone extraction. The exclusion of those under 18 or over 60 means 24 years		Experimental group: (Clindamycin 5-day group): 600 mg of clindamycin hydrochloride orally 60 min preoperatively, followed by 300 mg of clindamycin hydrochloride every 8 h for 5 days. N = 28 Control group: 600 mg placebo orally 60 min before surgery, followed by a dose of 300 mg placebo every 8 h for 5 days. N = 27		Ketoprofen 50 mg capsules to be taken in case of pain. The maximum daily dose was 200 mg. The patients were asked not to take any other medications during the trial		On a 4-degree scale, Trismus—facial swelling, submandibular lymphadenopathy on a 100 mm VAS body temperature, pain Alveolar osteitis (the clinical diagnosis of this complication was given in the case of the presence of a necrotic gray clot in a bare bone cavity, the fetor ex ore, accompanied by pain in this area) Follow-up period: on the first, second and seventh postoperative days		Experimental group: 2/28 Control group: 4/27 Not included in the study There is a single-dose group: patients receiving 600 mg of clindamycin hydrochloride orally 60 min preoperatively		9 did not register for the follow-up exam 3 were disqualified due to complications 2 resigned during the trial without giving any reason Side effects 3% of the participants who took a 5-day course of clindamycin developed gastric complications and were excluded from the trial	
Adde [34] 2012 Brazil Funding source: unspecified	RCT Age between 18 and 45 years. ASA type I with indication for the extraction of upper and lower third molars		Experimental group: Clindamycin 300 mg 4 times a day for 7 days. N = 23 Control group: no treatment. N = 24		Diclofenac 50 mg every 8 h for 3 days Paracetamol 750 mg at least 1 h before surgery and then every 6 h until pain stops		Postoperative infection: body temperature greater than 37.8C with no other perceptible causes, intraoral abscess with floating point of drainage, alveolitis, persistent severe pain or intensified pain 48 h after surgery and inflammation and / or erythema, and severe pain at from the seventh day accompanied by inflammation Follow-up period: Evaluation at 24 h, 48 ​​hours, 3 days and 7 days.		Experimental group: 0/23 postoperative infection Control group: 0/24 postoperative infection		No losses Side effects No complications of any kind were reported	
Kaposvári [37] 2017 Hungary No Funding	RCT lower third molar was removed Healthy patients 18 to 35 years mean age 24.78 year		Experimental group: 600 mg of clindamycin one hour before surgery. N = 14 (7 simple/7 complex) Control group: placebo. N = 18 (8 easy / 10 complex)		Diclofenac 50 mg, maximum 3 dose		Alveolitis Dissected wound Follow-up period: for a week. till the remove of the suture		Experimental group: 0/14 alveolitis and dissected wound 2/14 Control group: 2/18 alveolitis and 4/18 dissected wound		1 loss in the experimental group Side effects: wound separation, edema and lockjaw	
INTRAVENOUS CLINDAMYCIN IN THIRD MOLAR EXODONTIA												
Halpern [12] 2007 US supported in part by the Oral and Maxillofacial Surgery Foundation Research Grant and Massachusetts General Hospital (MGH) Center for Applied Clinical Investigation		RCT Patients requiring third molar extraction under intravenous sedation or general anesthesia in the outpatient setting in the office ´ Age range of patients is 17.7–31.5 years. Average of 25 years		Experimental group: penicillin solution (15,000 units per kilogram) or, in the case of subjects allergic to penicillin, clindamycin 600 mg intravenously 1 h before the intervention N = 0.60 N = . (clindamycin) 15 Placebo control group: solution (10 cc of 0.9% saline) administered intravenously within 1 h before the intervention N = 62		All subjects received preoperatively intravenous dexamethasone (8 mg) and 15% received intravenous antiemetic therapy (Ondansetron 2 mg)) 1 or 2 tablets of paracetamol (500 mg) and hydrocodone (5 mg) administered orally every 3 to 4 h		Dry socket: a new onset or increase in pain more than 36 h after the operation, with the blood clot at the extraction site as evidenced by exposed bone, gentle probing or irrigation of the wound that doubles the pain and relief significant pain after the operation Surgical site infection: visual evidence of frank purulence at one or more of the extraction sites and Gram stain demonstrating the presence of white blood cells Follow-up period: Evaluated on postoperative day 7 (range 5–14)		Experimental group: 0/15 postoperative infection Control group: 5/62 postoperative infection		1 loss in the control group 1 loss in the experimental group
TOPICAL CLINDAMYCIN IN THIRD MOLAR EXODONTIA												
Hamiti-Krasniqi [35] 2012 Kosovo Funding source: unspecified		RCT A split mouth Extraction of the right and left mandibular 3rd molar. Patients with health problems and those who received antibiotic therapy 14 days before surgery were excluded		Experimental group: 300 mg clindamycin mixed with 0.2 ml of saliva. Gelatamp hemostatic sponge is then inserted N = 0.60 molars Control group: nothing. N = 0.60 molars The patients were divided into smokers and non-smokers		Analgesic medication is given only in the case of post-extraction pain, specifying the side of pain		Dry socket Follow-up period: the next day, two days and day 5		Experimental group: 2/60 dry socket Control group: 19/60 dry socket		No loose to follow up
ORAL CLINDAMYCIN IN ENDODONTIC SURGERY												
Lindeboom [28] 2006 Amsterdam Funding source: unspecified		RCT Tooth with apical periodontitis with adequate root closure and coronal restoration		Experimental group: clindamycin 600 mg 1 h before incision N = 128 teeth Control group: placebo 600 mg 1 h before incision N = 128 teeth		0.2% chlorhexidine solution twice a day for 1 week		Infection: Purulent drainage from an incision or drain, serosanguineous drainage, and wound culture positive for a known pathogen, wound spontaneously dehisced or deliberately opened by surgeon when patient had fever or localized pain or tenderness, with positive culture of the wound Follow-up period: patients were evaluated at the 1st, 2nd and 4th week		Experimental group: 2 teeth / 128 infection Control group: 4 teeth / 128 infection		No loose to follow up

### Risk of bias in individual studies

**Fig. 2:**
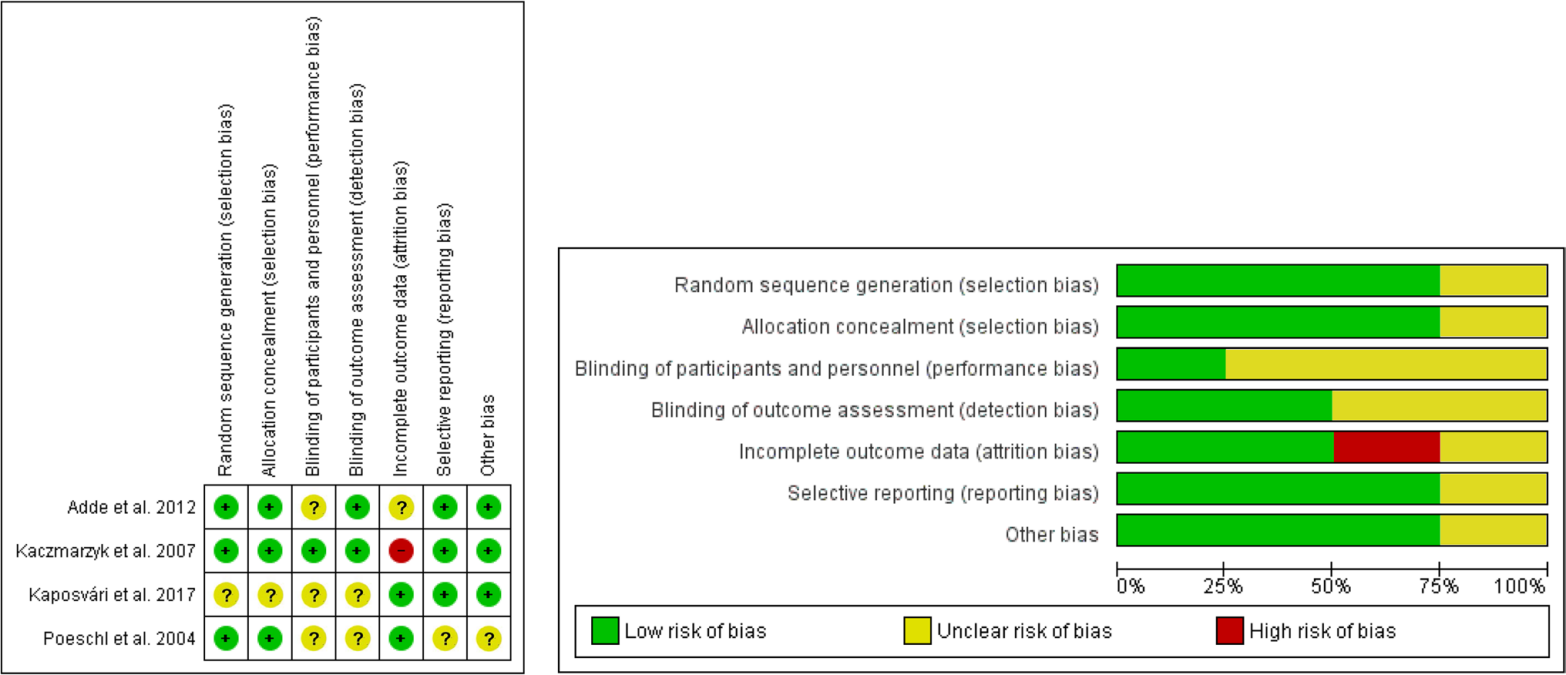
Risk of bias of included trials in quantitative analysis

The Cochrane Collaboration's tool was used to assess the individual risk of bias of each RCT included in quantitative analysis (Fig. 2). The bias in each study was analyzed using the recommended approach for assessing risk of bias in studies included in Cochrane reviews.

### Summary measures

The effectiveness of the treatment was assessed considering the relative risk (RR). The differences in incidences between the treatment and control groups or attributable risk were utilized to assess the clinical significance of the treatment with clindamycin. Furthermore, the number needed to treat (NNT) was calculated.

### Synthesis of results

The analysis was carried out using StataIC 13 (Stata-Corp LP, College Station, College Station, TX) and Review Manager (RevMan) 5.2 version (Copenhagen: The Cochrane Collaboration, 2012) software. We assessed the heterogeneity of the different studies using the I^2^ statistic. The overall relative risk, resulting from combining outcomes from the different studies, was calculated with inverse variance-weighted Mantel–Haenszel (MH) model. Empirical correction was used for the studies with zero effect sizes in one of their arms, and any studies with a zero effect size in both arms were excluded from the analysis.

## Results

### Study selection

We identified 540 records in both the databases and manual search (Fig. 1). After removing duplicates, 38 articles were selected for the full-text assessment. After full-text assessment, 7 were included in qualitative synthesis. First, all articles that did not analyze the infection clinically were excluded. Nine articles [38–46] studied bacteremia, three articles [47–49] studied the influence of clindamycin on the oral microbiome. Bulut et al. (2001) [50] studied the levels of the acute phase of proteins. One article [51] could not be found and it was excluded. Afterwards, the articles were classified according to the type of oral surgery in which the effectiveness of clindamycin was tested. Table 1 shows the studies that were included and those that were excluded with their reasons.

### Study characteristics

Table 2 shows the studied variables of the included studies: one study was performed on endodontic surgery and, six studies on third molar surgery. Hamiti-Krasniqi et al. (2014) [35], tested topical clindamycin in the prevention of dry socket, while Halpern and Dodson (2007) [12] used intravenous clindamycin (600 mg IV 1 h before surgery). Both studies showed lower infection rates in patients treated with clindamycin than in the placebo group. In the rest of the clinical trials, the treatment was with oral clindamycin, varying in their regimens and dosages. The follow-up period throughout the studies ranged from 1 to 4 weeks.

Only four trials allowed us to pool information on the effect of oral clindamycin in third molar extractions. For this reason, we decided to continue with a quantitative analysis testing the null hypothesis (H0), with a significance level of *p* = 0.05, that the preventive use of oral clindamycin is not effective in reducing infection in third molar surgery.

### Risk of bias within studies

Risk of bias of each study is presented in Fig. 2. Despite the fact that some studies were not of high quality and that they dealt with different doses, the quantitative analysis was perform including the four articles [31, 33, 34, 37] in which the efficacy of oral clindamycin in third molar surgery was studied.

### Summary measures

The four studies in which oral clindamycin was prescribed to prevent infectious complications after third molar extraction were the only included ones. The quantitative analysis involved 486 extractions, 245 of them treated with clindamycin and 241 from the control group (treated with placebo or with no treatment). There were 19 and 27 reported infection, dry socket or other events in respective group.

**Fig. 3:**
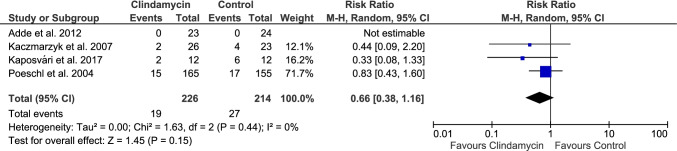
The Forest Plot diagram

The Forest Plot (Fig. 3) shows the graphic representation of the RR and 95% CI estimates performed with the samples of the 4 included studies. The overall RR extracted from all the studies indicated that there was no statistical benefit, and oral clindamycin may not be effective in the prevention of infectious complications after third molar extractions.

### Synthesis of results

The heterogeneity measured from the I^2^ test was 0 (*p* = 0.44), the null hypothesis of absence of heterogeneity between the results of the studies included in this meta-analysis could not be rejected. The Q statistic also supports the assumption of homogeneity between studies.

The overall RR, by using the Mantel–Haenszel (MH) method was found to be 0.66, with a 95% CI of 0.38 to 1.16, being non-statistically significant (*p* = 0,15). This range also included the value 1, indicating that clindamycin treatment may not prevent the development of infectious complications (dry socket, infection, or both conditions at the same time) following third molar extractions.

### Analysis of clinical significance

The NNT was 29 and it ranged between 12 and -57. This means that between 12 and infinity patients would need to be treated with oral clindamycin to prevent a single case of infection after third molars extraction. These results indicated that oral clindamycin may be ineffective in preventing infections following third molar extraction.

## Discussion

The principal findings of this systematic review and meta-analysis were the small number of studies available, focusing on the effect of prophylactic clindamycin in oral surgery procedures, despite being the antibiotic of choice in patients with hypersensitivity reactions to penicillins [12–14, 16, 33].

The quantitative analysis carried out on four studies that evaluated the effect of oral clindamycin in third molar extractions showed the ineffectiveness of clindamycin preventing infection complications.

Furthermore, the main weaknesses of this study lie in the small number of publications that could be included. Only seven clinical trials [12, 28, 31, 33–35, 37] met the inclusion criteria: six on third molar extractions, one in endodontic surgery [28] and no one on oral implant surgery. In the rest of oral surgical interventions [17–27, 29], the authors did not use a control group with placebo or without any treatment. This may be due to ethical reasons. Nevertheless, absence of a control group impedes the effectiveness assessment of the tested treatments.

Some RCT [13, 32] analyzed the preventive effect of amoxicillin, replacing it for clindamycin when the patient was allergic to penicillin. However, most studies did not specify the sample size of each antibiotic or the number of infected patients according to the antibiotic that was finally used.

Another aspect to take into account is the sample size of each study. In the quantitative analysis, the total number of extractions was 486: 245 treated with oral clindamycin and 241 belonging to the control group. In addition, we must not forget that each of the trials studied a different antibiotic prescription pattern.

Besides, the risk of bias of each of the studies must also cautiously considered. In fact, there were no signs indicating publication bias in the present review, yet there may be a possibility that small-sized and negative studies might not have been published.

Nevertheless, there may be important implications for clinicians emerging from the present study. Nowadays, there is no consensus on the need to prescribe preventive antibiotics in oral surgery such as third molar extractions or oral implant placements in healthy patients. Reviews and meta-analysis have been conducted by using mainly beta-lactam antibiotics for prophylaxis. In 2021 a Cochrane review [6] concluded that there was evidence that prophylactic antibiotics reduce the risk of infection, dry socket and pain, following third molar extractions and resulted in an increase in mild and transient adverse effects. However, due to the increasing prevalence of bacteria which are resistant to treatment by currently available antibiotics, clinicians should consider carefully whether treating 12 healthy patients with antibiotics to prevent one infection (NNT) is likely to do more harm than benefit [6].

Healthy patients allergic to amoxicillin are frequently treated preventively with clindamycin in oral surgery. In the present meta-analysis with oral clindamycin the NNT was 29 (ranging from 12 to -57). These results indicate that oral clindamycin may not only be ineffective in preventing infections after third molar extraction, but it may even have a negative effect. With the limitations of the study, published in 2021 [52] authors state that clindamycin has been associated with a significantly elevated risk of failure of dental implant, and an up to six times increased risk of infection after surgical implant placement. Immediate implants also had a 5.7 to 10 times higher risk of failure.

The NNT is only a part of the information required to make decisions. Therefore, when the clinicians prescribe antibiotics before and/or after oral surgery to prevent infectious complications, other factors such as costs, adverse effects, patient characteristics, and social priorities must also be considered. Recent evidence also implicates clindamycin with a higher adverse-effect profile than amoxicillin, and pseudomembranous colitis is a key adverse outcome of clindamycin with an incidence of 2 to 10% [16].

Educational programs, clinical guidelines, professionals and educators should promote the improvement of the use of prophylactic antibiotics in oral surgery. They should also attempt to reduce the possible gap between the antibiotic prophylaxis usage supported by scientific evidence and the real antibiotic prescriptions performed by professionals.

This review highlights the need for further research focusing on clindamycin, with different dosages and adverse drug reactions, particularly in those surgical procedures where it is frequently prescribed as a prophylactic treatment.

It would also be interesting to review the efficacy of other antibiotics such as clarithromycin, azithromycin and metronidazole that are also used as preventive treatment in oral surgery procedures in patients allergic to amoxicillin. Clarithromycin is another acceptable penicillin substitute. This drug has a more limited spectrum of activity than clindamycin but has some advantages over erythromycin. Clarithromycin is effective against facultative anaerobes and some of the obligate anaerobic bacteria. Metronidazole is a synthetic antibiotic that is highly effective against obligate anaerobes but is not effective against facultative anaerobic bacteria.

In conclusion, there was not enough evidence to evaluate the effectiveness of preventive clindamycin in oral surgical interventions other than third molar extraction. The null hypothesis that oral clindamycin is not effective in preventing infection in third molar surgery regardless of the dosage used may be accepted.

## Data Availability

The data that support the findings of this study are available from the corresponding author upon reasonable request.
